# Supplementary material for: Stability and sensitivity of interacting fermionic superfluids to quenched disorder
Source: Nat Commun. 2024 Oct 28;15:9292. doi: 10.1038/s41467-024-51903-8 (PMC11519488; doi:10.1038/s41467-024-51903-8)
Supplement: Supplementary file 1 — Supplementary Information [file 41467_2024_51903_MOESM1_ESM.pdf]

Supplementary Material for Stability and sensitivity of interacting fermionic superfluids to quenched disorder

Jennifer Koch,[1,2] Sian Barbosa,[1] Felix Lang,[1] and Artur Widera[1]

[1] *Department of Physics and Research Center OPTIMAS, RPTU Kaiserslautern-Landau, 67663 Kaiserslautern, Germany*

[2] *TOPTICA Photonics AG, Lochhamer Schlag 19, 82166 Gräfelfing, Germany*

(Dated: August 13, 2024)

In this supplemental material, we present further details on the experimental setup and methods (see Supplementary Note 1) and provide additional information on the density response to disorder quenches (see Supplementary Note 2) and open-system disorder quenches (see Supplementary Note 3).

SUPPLEMENTARY NOTE 1: DETAILS ON THE EXPERIMENTAL SETUP AND METHODS.

A. Extraction of the maximum aspect ratio

The aspect ratio is the ratio of the width of the clouds in the axial and radial directions, where the width is extracted from the absorption images. We integrate the density distributions in the imaging plane separately along the x and y directions to obtain two one-dimensional density profiles. The width of the cloud is determined as the fitting parameter of the 1D density profile for each direction. However, the density profiles of a mBEC and a UFG are generally different, and the Thomas-Fermi density profiles of a mBEC and a UFG do not have the same exponent. To avoid any method-depending systematics and to obtain a homogeneous evaluation method when analyzing the density profiles along the crossover, we employ the same fitting profile for all density distributions measured at various interaction parameters. This is supported by Supplementary Fig. 1, where the different fitting functions are compared and show only small differences. Thus, we take the profiles that are suitable for a BEC as fit functions for the whole crossover. After expansion, the radial direction is the long axis of the cloud through inversion of the aspect ratio.
In the x direction, we apply a 1D Thomas-Fermi profile n_{TF} [1]

$$n_{\mathrm{TF}} \propto \left(1 - \frac{i^2}{r_{\mathrm{TF}_i}^2}\right)^2, \tag{S1}$$

with r_{TF_i} the radius in i-direction. Besides that, in the y direction, the density shows a sharp peak and a non-neglecting background, which makes it difficult to fit a Thomas-Fermi profile. Therefore, this direction is fitted with a 1D-bimodal fit $n_{\mathrm{bimodal}} = n_{\mathrm{G}} + n_{\mathrm{TF}}$. The background is fitted with a Gaussian 1D-profile n_{G} given by

$$n_{\mathrm{G}} \propto e^{-\frac{i^2}{2s_{i_{\mathrm{G}}}^2}}, \tag{S2}$$

with the width $s_{i_{\mathrm{G}}}$. We extract the full width at half maximum (FWHM) of the total fitted 1D-density profiles for both directions. The aspect ratio is the ratio of the FWHM of the x direction by the one of the y direction. By varying the expansion time d, we measure a change in the aspect ratio. The maximum value quoted in the main text is extracted as the mean from the three highest ratios during the expansion evolution, and their standard deviation is indicated by the error bars.
For comparison, on resonance, the 1D Thomas-Fermi density profile n_{TFU} is [2]

$$n_{\mathrm{TFU}} \propto \left(1 - \frac{i^2}{r_{\mathrm{TFU}_i}^2}\right)^{5/2}, \tag{S3}$$

with the radius r_{TFU_i} in i-direction. Supplementary Figure 1 shows fits of the measured density profiles and compares a bimodal fit (Eq. S1 & S2) with the Thomas-Fermi fit on resonance (Eq. S3) for clouds at resonance. Both versions fit the data well.

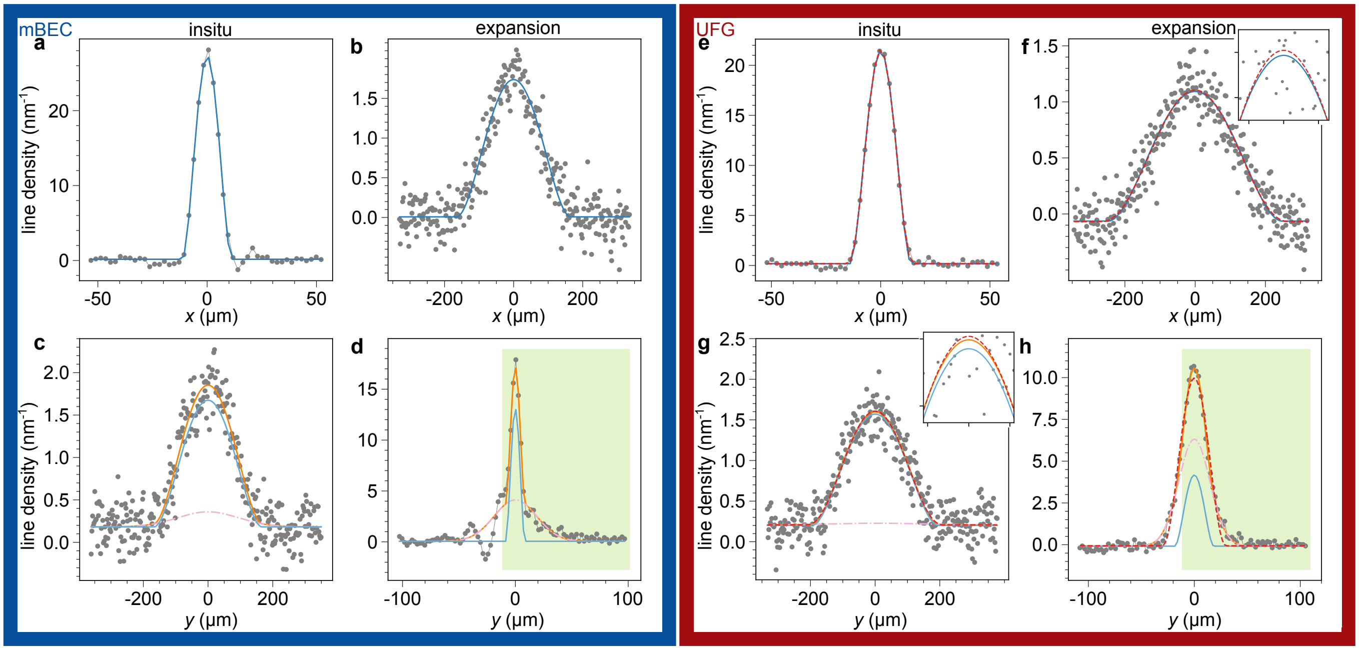

Supplementary Fig. 1. **Comparison of different fit functions to in situ and expansion density profiles.** Measured line density (gray points) in dependence of the x and y direction, once in the mBEC regime at 763.6 G and once at resonance at 832.2 G. (a,b) mBEC, in situ and expansion in dependence of the x direction, fit to the data with a Thomas-Fermi fit in the mBEC regime (solid blue line). (c & d) mBEC, in situ and expansion in dependence of the y direction, fitting to the data with a bimodal fit (solid orange line). The bimodal fit consists of a Thomas-Fermi fit (solid blue line) and a fit with a Gaussian profile (rose dashed-dotted line). (e & f) At unitarity, in situ and expansion in dependence of the x direction, fit to the data with a Thomas-Fermi fit in the mBEC regime (solid blue line) and with one of a UFG (red dashed line). (g & h) At unitarity, in situ and expansion in dependence of the y direction, fit to the data with a bimodal fit (solid orange line). The bimodal fit consists of a Thomas-Fermi fit in the mBEC regime (solid blue line) and a fit with a Gauss profile (rose dashed-dotted line). For comparison, a fit with a Thomas-Fermi fit at resonance (red dashed line). d) & h) show the line densities in y direction by expansion of the cloud. The measured line density is only fitted in the area marked with the green background and the whole cloud is inferred. Due to systematic imaging errors, it is difficult to fit the whole range. At resonance, both fit-methods fit well with the data.

B. Measuring particle losses versus trap depth

We adjust the atom losses induced by the disorder field through different compression values of the trap. A lower ODT laser power leads to a smaller compression and a lower trap depth, which leads to larger atom losses in a disorder quench and vice versa. For a sufficiently large compression of the trap, we measure no atomic losses that are caused by the speckle field. Supplementary Figure 2 shows the mapping between the power of the ODT laser after compression and the remaining particles in the trap. The measurements are performed for the mBEC regime at 763.6 G and on resonance at 832.2 G. As noted in the main text, the disorder strength of the speckle potential is twice as much for molecules compared to single atoms for the same laser power. Hence, for the mBEC regime, the speckle-laser power is set to 5 W and for the unitary regime to 10 W to get the same effective disorder potential. As a reference, the atom number is also measured without a disorder field for both regimes. For an identical trap depth, the remaining particles in the trap are equal in both regimes. In addition, we have investigated the losses for two different instantaneous disorder quenches, one when switching the disorder field on and one when switching it off. The amount of atoms that leave the trap is higher for a quench into the disorder field compared to a quench out of the disorder field, where we introduce the speckle potential adiabatically rather than instantaneously (see Supplementary Fig. 2). Moreover, we have measured similar loss curves for the other magnetic field strengths used. No losses occur for all applied magnetic fields for an ODT laser power of 80 mW.

For these measurements, as for the measurements presented in the main text, the evaporation of the cloud takes place at 763.6 G, and the power of the ODT laser is lowered to 8 mW. Subsequently, the cloud is held for 250 ms before the trap is compressed. The power of the ODT laser and hence the trap compression is set to a selected value. As the next step, the magnetic field is ramped to the desired value in a duration of 200 ms. Subsequently, the cloud is held in the trap for 30 ms before switching on the disorder speckle laser. Lastly, the cloud is imaged in situ by absorption

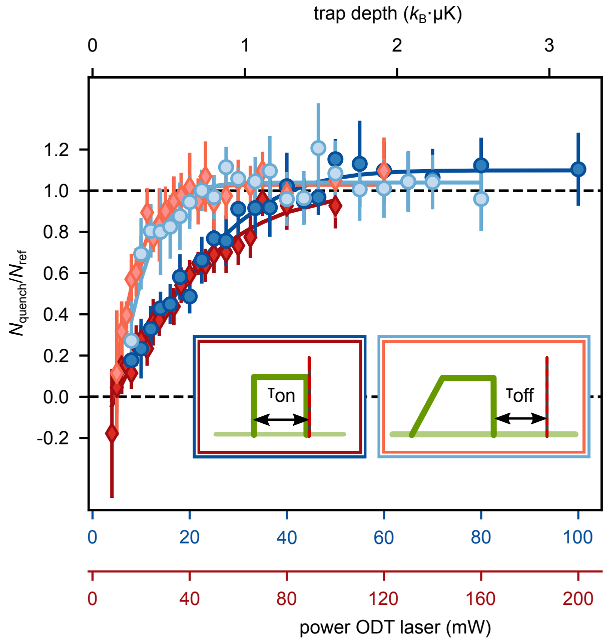

Supplementary Fig. 2. **Mapping losses.** Mapping of remaining atoms $N_{\mathrm{quench}}/N_{\mathrm{ref}}$ in the trap after quenching the disorder field as a function of the power of the ODT laser for the compression. N_{quench} (N_{ref}) is the atom number with (without) disorder field. For the unitary regime (832.2 G), a speckle power of 10 W (red, diamonds) and for the mBEC regime (763.6 G) a speckle power of 5 W is applied (blue, circles). The data points for the decay (dark) and the ones for the revival (light) are shown. The error bars are calculated by the error propagation of the standard deviation of five repetitions. The solid lines are fits with an error function. For comparison, the power of the ODT laser is mapped to the trap depth because this is different for molecules and atoms for the same powers (see red and blue axis). The left (right) inset shows the disorder pulse shape for the decay (revival) of the quantum properties. The imaging (vertical red line in inset) takes place in situ after an expansion time $d = 0$.

imaging. The atom number is determined by summing up the intensity of the picture pixel-wise.

C. Hydrodynamics from quantum to classical regimes

In order to compare the maximum aspect ratio of quantum gases after quenching the disorder potential with that of a gas above the critical temperature, we record the hydrodynamic expansion of a thermal gas without disorder. The temperature of the gas is adjustable via the final laser power of the ODT during evaporation. The power of the ODT laser at the end of the evaporation is varied between 8 mW and 350 mW. Afterward, the trap is compressed by increasing the power of the ODT laser. To avoid further evaporation and have the same conditions for the different powers at the end of the evaporation, the power for the compression is set to 400 mW for all varied powers. At an ODT laser power of 350 mW at the end of evaporation, the atomic cloud has a temperature of about 1200 nK. We measure the maximum aspect ratio of the cloud by expansion for two magnetic field strengths, 763.6 G and 832.2 G (see Supplementary Fig. 3). In both regimes, we see a decrease in the maximum aspect ratio with increasing power, i.e., temperature until it reaches a final state, a thermal gas, exhibiting purely classical hydrodynamics. At this final state, there is no long-range phase coherence present. For the UFG, the aspect ratio of the final, thermal state is higher than that of the mBEC. We attribute this to stronger interactions, leading to stronger collisional hydrodynamics. Due to the higher ODT power of 400 mW (power for the compression of values in Supplementary Fig. 3) compared to 80 mW (shown in Fig. 2(a) and Fig. 3(c) without losses), the aspect ratios are not comparable since the power effects the trap frequencies. Therefore, the aspect ratio of the thermal gas at 400 mW is adjusted to the one in Fig. 2(a) and Fig. 3(c) at 80 mW. It is scaled according to $\sqrt{\omega_{80\,\mathrm{mW}}/\omega_{400\,\mathrm{mW}}}$, where ω is the geometric mean of the trap frequencies in the radial directions. This follows from the ideal-gas condensate, where the expansion radius is proportional to the square-root of the trap frequency [3]. In addition, the thermal gas aspect ratio data for both interaction regimes in Fig. 1(d), measured at an ODT laser power of 400 mW, are scaled similarly, since the ideal time evolution is computed for 80 mW as for the degenerate regime.

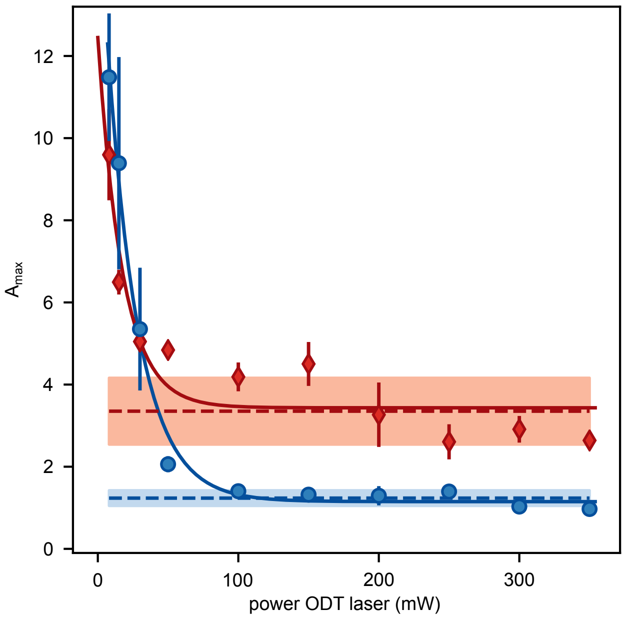

Supplementary Fig. 3. **Expansion of a thermal gas.** Maximum aspect ratio $A_{\max}$ as a function of the power of the ODT laser at the end of the evaporation. Shown are experimental data of a mBEC at 763.6 G (blue) and a UFG at 832.2 G (red). The experimental data are fitted with an exponential decay function (solid lines). The final state is fitted with a constant (dashed line) by the last six measured values. The fit uncertainties are the blue and red-filled areas around the dashed lines.

SUPPLEMENTARY NOTE 2: DENSITY RESPONSE TO DISORDER QUENCHES

The disorder potential leads to a spatial density variation of the cloud. For every parameter set used to measure the hydrodynamic response of quantum gases, we have also measured the density response. The difference between probing the density or the hydrodynamic response is the expansion time d. The density variation is probed by in-situ ($d = 0\,\mathrm{ms}$) absorption images of the column-integrated density distribution $n(x, y)$ of the atomic cloud while for probing the hydrodynamic response the expansion time is in the order of $d = 10\,\mathrm{ms}$. The evaluation procedure of the absorption images is taken from [4]. To extract the disorder-induced perturbation of the cloud, we first fit a 2D Thomas-Fermi profile [3]

$$n_{2\mathrm{D}} \propto \left(1 - \frac{(x - x_0)^2}{r_y^2} - \frac{(y - y_0)^2}{r_x^2}\right)^{3/2}, \qquad \text{(S4)}$$

which is valid in the mBEC regime. For a UFG, the profile should be distinguished from a mBEC in its power law. However, as discussed in the case of hydrodynamic expansion above, we apply the evaluation described for the mBEC regime for all data in order to obtain a homogeneous analysis. The fitted smooth 2D Thomas-Fermi profile is subtracted from the measured data, leaving only the density variations around the mean. The density perturbation is quantified via the fragmentation α, see Ref. [4], defined as

$$\alpha = \sqrt{\langle \Delta n^2 \rangle - \langle \Delta n \rangle^2}, \qquad \text{(S5)}$$

with $\Delta = n - n_{\mathrm{fit}}$, where n_{fit} is the fitted 2D Thomas-Fermi profile of the measured density distribution n. Further, the brackets denote averaging over all pixels, where $n_{\mathrm{fit}} > 0$. Due to fluctuations already without any disorder field $\alpha \neq 0$ [4]. In contrast to the hydrodynamic expansion, the fragmentation occurs and recovers on similar time scales. Moreover, the density distribution becomes completely smooth, i.e. fragmentation completely vanishes after disturbing the cloud (see Supplementary Fig. 4(a)). This is independent of the interaction strength, the powers of the laser (5 W and 10 W), which creates the disorder potential, and even in an open system with significant atom/molecule losses (see Supplementary Fig. 4 (d) and 5(d)). We interpret this as a consequence of local transport processes occurring,

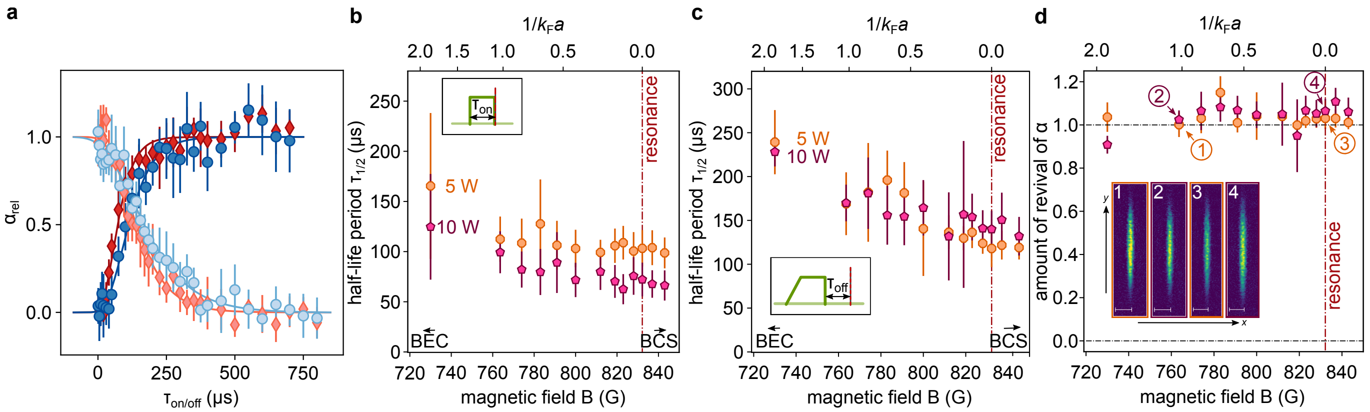

Supplementary Fig. 4. **Density response to disorder quenches.** (a) Decay (dark blue and dark red) and revival (light blue and light red) of the fragmentation α, normalized to the respective maximum of the fit function ($\alpha_{\rm rel}$), as a function of the disorder pulse duration $\tau_{\rm on}$ or the revival time $\tau_{\rm off}$. Shown are the experimental values of a mBEC at 763.6 G (blue points) and a UFG at 832.2 G (red diamonds), where each data point is averaged from five measurements, and its standard deviation corresponds to the error bar. The solid lines are fits with a Gompertz function. Further, the offset o, which is extracted from the fit, is subtracted from the data and the fit to obtain the fragmentation, which is only caused by the disorder field. (b) Interaction dependence of the density response to quenches into disorder. Half-life period $\tau_{1/2}$ as a function of the magnetic field. As for the expansion dynamics, the half-life period decreases from the mBEC side towards the resonance. The inset shows the pulse shape for the decay measurement. The cloud is immediately imaged in situ after the disorder pulse (vertical red line). Error bars are determined via the fit uncertainties (see Eq.S7). (c) Interaction dependence of the density response to quenches out of disorder. Half-life period $\tau_{1/2}$ as a function of the magnetic field. The half-time period of the density variation decreases with decreasing the interaction parameter. The inset shows the pulse shape for the revival measurement. The cloud is imaged in situ (vertical red line) after the revival time $\tau_{\rm off}$. (d) Amount of revival of the fragmentation α in dependence of the magnetic field B. The fragmentation fully recovers for 5 W (orange) and even for 10 W (purple) power of the disorder laser. The inset shows in situ absorption pictures when the fragmentation reaches its highest value due to the disorder field.

where we cannot distinguish classical or quantum contributions.
For extracting the half-life period, the fragmentation is fitted with a Gompertz function g [4, 5]

$$g(t) = a\,e^{-e^{-t/c}/b} + o, \tag{S6}$$

with the fit parameters a, b, c and o. The half-life period $\tau_{1/2}$ is calculated by

$$\tau_{1/2} = -c\,\ln(b\,\ln(2)). \tag{S7}$$

The amount of the revival of the fragmentation is calculated from the fit parameters. The minimal fragmentation $\alpha_{\rm min}$ of the revival (quench out of) is set in relation to the minimal fragmentation of the decay measurements (quench into). For the decay measurement, the Gompertz function is calculated for $t = 0$ ($\alpha_{\rm min} = a\,e^{(-1/b)} + o$). For the revival measurement, t is considered in the limiting case towards infinity ($\alpha_{\rm min} = a + o$).
In a direct comparison of the half-life periods between a mBEC and a UFG, we measure that the new final state, for the decay measurement, is assumed faster for a UFG (UFG: $\tau^{\rm on}_{1/2} = (72 \pm 17)\,\mu$s, mBEC: $\tau^{\rm on}_{1/2} = (112 \pm 23)\,\mu$s). We attribute this to a larger interaction strength and, thus, increased collision rate. The same can also be seen with the revival (UFG: $\tau^{\rm off}_{1/2} = (140 \pm 21)\,\mu$s, mBEC: $\tau^{\rm off}_{1/2} = (169 \pm 36)\,\mu$s). Over the BEC-BCS crossover, we see a decrease in the half-life period with increasing magnetic field (see Supplementary Figs. 4(b) and (c)). This can be attributed to the larger scattering length, where local particle scattering and transport mechanisms may be enhanced.
By directly comparing the hydrodynamics and the density response, we see that the decay of the hydrodynamics for the mBEC an the UFG is one order of magnitude faster than the density response, as has been seen in Ref. [4]. This can be understood intuitively for the mBEC regime, where the quenched disorder potential imprints a random local phase on the many-body wavefunction. This leads to quick dephasing and decay of long-range coherence. Once local phase differences have been established, this phase difference drives local currents that lead to the emergence of the density variation of fragmentation at a later time. Therefore, the density response is delayed compared to the hydrodynamic decay.

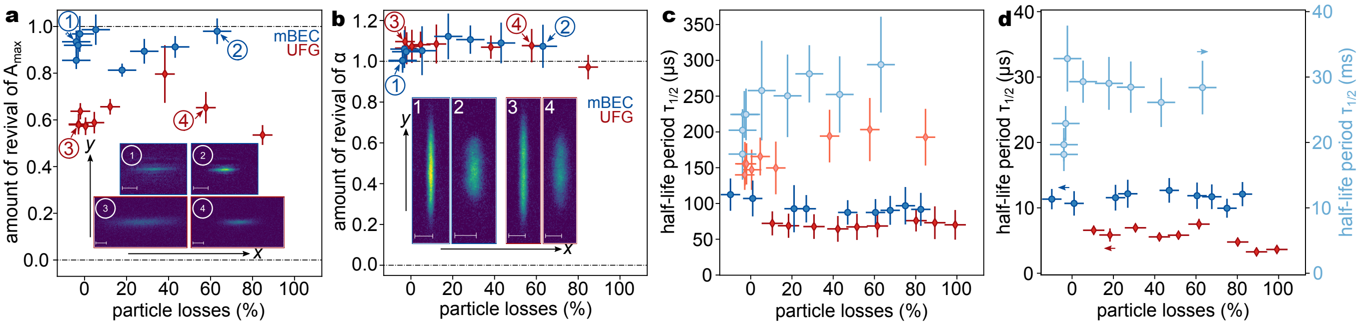

Supplementary Fig. 5. **Quench in an open system.** (a) Amount of revival of the maximum aspect ratio $A_{\rm max}$ as a function of atom losses (see Methods), which are controlled via the trap depth. A mBEC, blue circles, (UFG, red diamonds) at 763.6 G (832.2 G) is perturbed with 5 W (10 W) disorder laser power. The aspect ratio after a long revival time ($\tau_{\rm off} > 200\,$ms) is taken as the maximum achievable quantum hydrodynamic in equilibrium taken from a fit to eq. (5) as in panel Fig. 3(a) and normalized to the maximum value without disorder, see Methods. The insets show absorption images for the indicated parameters. (b - d) Density response for disorder quenches in an open system. Particle losses for the mBEC (UFG) at 763.6 G (832.2 G) for a power of the disorder laser of 5 W (10 W) (b) Amount of the revival of the fragmentation. The minimum of fragmentation of the revival measurement is set in ratio to the minimum of the fragmentation $\alpha_{\rm min}$ of the decay measurement. Values are extracted from the fit parameters of the Gompertz-fit, and the error bars are calculated via error propagation of the fit uncertainties. (c) Half-life period $\tau_{1/2}$ for the decay (dark) and revival (bright) of density variation for the unitary (red) and mBEC (blue) regime as a function of provoked atom losses. Revival takes roughly a factor of two longer than the decay of the density. The decay time for expansion and fragmentation is unaffected by losses, but the revival time increases with particle losses. (d) Half-life period $\tau_{1/2}$ for the decay (dark) and revival (bright) of the aspect ratio for a UFG (red) and mBEC (blue) as a function of provoked particle losses. Only the mBEC is shown for the revival because the UFG does not fully revive.

SUPPLEMENTARY NOTE 3: OPEN-SYSTEM DISORDER QUENCHES

Furthermore, we have also studied the response for dissipative quenches (see Supplementary Fig. 5(a-d)). Supplementary Figure 5(a) shows the amount of revival of the aspect ratio as a function of the particle losses that occurred. For the density response, even with losses, the fragmentation fully revives for a mBEC and a UFG (see Supplementary Fig. 5(b)). The losses influence the revival time, which increases for both interaction regimes with higher particle losses (see Supplementary Fig. 5(c)). The half-life period for the decay seems to be unaffected by the particle losses. This might be explained by a modified density after loss have occurred. The local collisions that re-smooth the density occur at a reduced rate when the density is reduced, resulting in a longer time to reach equilibrium. Further, the same is observed for the half-life period by the hydrodynamic response of the mBEC (see Supplementary Fig. 5(d)). As in [4], the half-life period for the revival of a mBEC takes some orders of magnitude longer than the decay. For reduced density, the time for reaching an equilibrium quantum gas is reduced. We attribute this to the fact that the local collision rate sets the time for decay of Bogoliubov excitations.

SUPPLEMENTARY REFERENCES

[1] S. Jochim, *Bose-Einstein Condensation of Molecules*, Ph.D. thesis, Leopold-Franzens-Universität Innsbruck, Supervisor: Rudolf Grimm (2004).

[2] M. Bartenstein, A. Altmeyer, S. Riedl, S. Jochim, C. Chin, J. H. Denschlag, and R. Grimm, Crossover from a Molecular Bose-Einstein Condensate to a Degenerate Fermi Gas, Phys. Rev. Lett. **92**, 120401 (2004).

[3] D. S.-K. W. Ketterle, D.S. Durfee, Making, probing and understanding Bose-Einstein condensates, arXiv https://doi.org/10.48550/arXiv.cond-mat/9904034 (1999).

[4] B. Nagler, S. Barbosa, J. Koch, G. Orso, and A. Widera, Observing the loss and revival of long-range phase coherence through disorder quenches, Proceedings of the National Academy of Sciences **119**, e2111078118 (2022).

[5] E. W. Weisstein, "Gompertz Curve." From MathWorld–A Wolfram Web Resource (accessed: 05.06.2023), `https://mathworld.wolfram.com/GompertzCurve.html`.
